# The Experiences of Healthcare Services and Ageing Among Older Turkish Immigrants: A Qualitative Study

**DOI:** 10.1111/jan.16763

**Published:** 2025-01-22

**Authors:** Süleyman Şahin, Büşra Nur Temür, Selma Öncel, Nilgün Aksoy, Anne‐Sofie Helvik

**Affiliations:** ^1^ Faculty of Health Sciences, Department of Public Health Nursing Kafkas University Kars Türkiye; ^2^ Faculty of Nursing, Department of Surgical Nursing Akdeniz University Antalya Türkiye; ^3^ Faculty of Medicine and Health Sciences, Department of Public Health and Nursing Norwegian University of Science and Technology (NTNU) Trondheim Norway; ^4^ Faculty of Nursing, Department of Public Health Nursing Akdeniz University Antalya Türkiye; ^5^ Norwegian National Centre for Ageing and Health Vestfold Hospital Trust Tønsberg Norway

**Keywords:** healthcare services, immigrant, nursing, older adult, public health, qualitative study

## Abstract

**Aims:**

The aim of this study is to explore patterns of the lived experiences of first‐generation Turkish immigrants (≥ 60 years) living and ageing in Norway regarding their experiences with healthcare services and ageing.

**Design:**

This study used a qualitative study.

**Methods:**

The sample consisted of 17 individuals aged 60 and above who were of Turkish origin, and immigrated to and living in Norway. All participants resided in the same city in the middle part of Norway. Individual face‐to‐face interviews were conducted between February and June 2023. All transcripts were examined using reflexive thematic analysis.

**Results:**

Two main themes and five subthemes were identified. The first theme was ‘Utilization of the healthcare service to their best’, with the subthemes: (a) ‘Communication through a translator: Expressing health problems’ and (b) ‘From physician to physician: Seeking a second opinion in health’. The second theme was ‘Being born in Türkiye: Aging in Norway’, with the subthemes: (a) ‘Between two worlds: Efforts to establish balance’, (b) ‘Family ties and care preferences: Understanding the care preferences’ and (c) ‘Two cultures, one life: Lifestyles’.

**Conclusion:**

This study reveals the experiences Turkish immigrants have with the healthcare service and ageing while living in Norway and balancing between two cultures. These findings offer a valuable perspective for healthcare providers and social workers and offer insight relevant to developing a cross‐cultural healthcare service programme.

**Impact:**

This study could provide a fundamental step towards understanding older Turkish immigrants and how healthcare services fit their needs. The results revealed Turkish individuals' experiences with ageing and healthcare services, including the use of translator services and general care preferences. The development of more inclusive support programmes for ageing immigrant populations may have far‐reaching impacts on individuals' ability to live healthy and meaningful lives.

## 1 Introduction

The world is currently experiencing a period in which the proportion of older people in the total population is increasing due to reasons such as longer lifespans and falling birth rates (King et al. 2016). This demographic change presents both challenges and opportunities. At the same time, people are migrating across borders more frequently for reasons such as economic opportunities, family reunification or the escape from conflicts and environmental problems. These global migration movements have an impact on many parts of the world (Agergaard et al. 2023). Due to migration and longer lifespans, we now have a new group of inhabitants in many European countries, that is, older immigrants. This group includes both individuals who immigrate to another country when they are young and grow old there, as well as individuals who migrate later in life to join their families or seek a different lifestyle in retirement (King et al. 2016). Ageing immigrants face different challenges that distinguish them from both their nonimmigrant peers in the host countries and the younger immigrant population (Wilding and Baldassar 2018). While immigrants adapt to the new social, cultural and political environment in the countries they move to, they are still often affected by sociocultural, economic and political factors in their countries of origin (Castañeda et al. 2015). Because adult immigrants often tend to preserve the cultures they inherit, they may experience intercultural struggles due to differences in values and lifestyles (Jacobsen et al. 2023). Language barriers, cultural disparities, difficulties accessing healthcare and differences in health literacy, can make it difficult to interact effectively with healthcare providers and receive appropriate care (Cramm and Nieboer 2018). Additionally, the process of integrating into a new community can be challenging, as it may be difficult for immigrants to find individuals with similar backgrounds, values and beliefs, which can impact their sense of identity and belonging (Harrison, Harrison, and Shaffer 2019). Although the link between health and cultural integration is not fully understood, it is generally recognised that effective integration is an important and necessary element of good health (Haj‐Younes et al. 2022).

It is important to better understand the health needs of Turkish immigrants in Norway and take this information into account in health policies. This study sheds light on current multifaceted issues by emphasising the importance of understanding the experience of Turkish immigrants' regarding healthcare use and ageing in Norway.

## 2 Background

The number of international migrants worldwide is estimated to reach 272 million people, approximately 3.5% of the world's population. Although the majority of people still live in the country they were born in, many people migrate to other countries, often away from their geographical region of origin (McAuliffe and Triandafyllidou 2021). Norway's population consists of a wide variety of immigrants, accounting for 16% of the total population. These immigrants stem from various cultural and socioeconomic backgrounds and have residence periods of varying length (Statistic Norway 2023). One of the largest and oldest immigrant communities in Norway is the Turkish community. Increasing demand for workers led to the first major wave of Turkish immigrants to Norway in the late 1960s (Rieger, Terragni, and Czapka 2021). Since then, Turkish people have migrated to Norway to reunite with family members, complete higher education or seek asylum (Rogstad 2009). As of January 2023, there were 14,295 Turkish immigrants in Norway, and this number includes Turkish parents born abroad. There were also 7597 children of Turkish immigrants with two Turkish parents (Statistic Norway 2023). Since the number of immigrants is increasing, ageing will become one of many important issues for the Norwegian government in the years to come.

All Norwegian citizens, residents of more than 6 months and registered refugees have access to public primary and secondary healthcare services (Herrero‐Arias and Diaz 2021). It is important that immigrants are able to use these services effectively. However, Turkish immigrants face many difficulties in this process. These challenges include language barriers, legal status, cultural beliefs regarding health and disease and familiarity with the host country's healthcare system (Rieger, Terragni, and Czapka 2021). These factors affect to which degree immigrants are able to benefit from health services, which varies greatly depending on age, gender, socioeconomic status and length of stay in the host country (Biyikli Gültekin 2017; Cramm and Nieboer 2018). Therefore, it is important to address whatever experiences Turkish immigrants have regarding the use of healthcare services to ensure that these services are accessible.

Cultural adaptation of Turkish immigrants is complex and involves integrating their traditional culture with the culture of the host country. This adaptation process includes learning a new language, understanding and using a different healthcare system, as well as combining traditional health beliefs with modern medical practices (Cokluk and Tokovska 2023). How quickly and easily Turkish immigrants adapt to this process may vary depending on various factors such as community support, family structure, education level and personal resilience (Özbek, Bohn, and Berntsen 2021). Many older immigrants maintain deep ties to their countries of birth and travel frequently between their home countries and the new countries where they live (Wilding and Baldassar 2018). Turkish immigrants have generally been found to show a strong attachment to their own ethnic community, which may affect their integration into the host society (de Vroome, Verkuyten, and Martinovic 2014). Some studies have investigated the experiences of older Turkish immigrants in European countries (Cramm and Nieboer 2018; De Tavernier and Draulans 2018; Ten Kate, Bilecen, and Steverink 2021). However, none of these studies focused specifically on experiences with the healthcare service and ageing. Furthermore, none of them were based in Nordic countries, which have a public healthcare service. Thus, our study addresses the gap in knowledge regarding how older Turkish immigrants experience healthcare services and ageing in Norway.

## 3 The Study

### 3.1 Aim

The aim of this study is to explore patterns of the lived experiences of first‐generation Turkish immigrants (≥ 60 years) living and ageing in Norway regarding their experiences with healthcare services and ageing.

## 4 Methods

### 4.1 Design

This study used a qualitative design performing individual interviews in the period between February and June 2023. The choice of reflexive thematic analysis allows for an in‐depth exploration of the data, guided by the author's expertise and knowledge, while emphasising the reflexivity inherent in the method (Näsström et al. 2023).

### 4.2 Study Setting and Recruitment

The sample consisted of Turkish individuals 60 years and above living in Norway. A total of 17 participants, 10 women and seven men, were included. All participants resided in and around the city in the middle part of Norway. Participants were selected using the ‘snowball sampling method’ to identify and recruit participants with specific characteristics or experiences. The aim was to ensure good participant diversity, encompassing both genders and various social backgrounds. Initially, an interview was conducted with an individual who met the inclusion criteria in a social setting where older Turkish immigrants often gather. This initial participant was subsequently asked to refer others within their social networks who also fit the criteria. This method facilitated access to a population that might otherwise have been challenging to reach. Criteria for inclusion in this study were being ≥ 60 years old, knowing and/or speaking either Turkish or Norwegian, having immigrated from Türkiye to Norway and having obtained Norwegian citizenship and being a resident of Norway at the time of the study. The exclusion criterion was having significant cognitive problems or language barriers in both Turkish and Norwegian, which could have made it difficult for participants to express their experiences during the interviews.

### 4.3 Data Collection

The interviews were conducted in quiet and comfortable places chosen by the participants, so they could express themselves freely. For male participants, interviews took place in a quiet part of a local cafe where Turkish men often gather to socialise. For female participants, some interviews were held in the women's area of a mosque, while others were conducted in their homes. These locations provided a familiar and private space for the participants to share their experiences openly. During the interviews, there was no one in the room except the researcher and the participant. Participants' data were collected through face‐to‐face interviews using a semi‐structured interview guide (Table S1). A guide was developed by the authors based on the aim, previous research literature and taking into account clinical experience and cultural identity (Mbanya et al. 2019; Tschirhart, Diaz, and Ottersen 2019). Each interview lasted a minimum of 14 and a maximum of 34 min. Participants were asked which language they preferred for the interviews. All participants chose their native language, Turkish. If anyone had preferred to conduct the interview in Norwegian, the last author would have conducted those interviews. The interviews were conducted by first and second authors, both of whom are PhD candidates with substantial experience and training in qualitative research methodologies, and both had previously conducted qualitative interview studies. The researchers reflected on their perspectives and assumptions before and after each interview, focusing on listening to participants' narratives and experiences. All interviews were audio recorded with participants' informed consent to ensure an accurate and complete registration of their statements. The audio‐recorded interviews were transcribed verbatim. For quality assurance, the transcripts were subsequently reread while listening to the audiotapes to ensure the accuracy of the transcripts. The data collection ended when duplications began to occur frequently, and no new information was received. After each interview, the reflexive sign was converted into field notes. These notes included observations about the interview setting, participants' nonverbal cues and any reflections responses or reactions during the interview. Individual interviews were conducted in person between February and June 2023. The choice of reflexive thematic analysis allows for an in‐depth exploration of the data, guided by the author's expertise and knowledge, while emphasising the reflexivity inherent in the method (Näsström et al. 2023).

### 4.4 Data Analysis

The data were analysed in line with the six‐phase reflexive thematic analysis method described by Braun and Clarke (2021). In the first step, all Turkish audio recordings were transcribed verbatim in Turkish by first and second authors and verified. Since last researchers did not speak Turkish, the transcripts were translated into Norwegian using a professional translation tool (Microsoft Translator at Norwegian University of Science and Technology—NTNU) to enable the last author to read and analyse the data in her native language. Team discussions and analysis meetings were conducted in English, the common language of the research team. When ambiguities arose, the original Turkish transcripts were consulted to ensure accuracy and data integrity. In the second step, initial codes for the data sets were developed separately by first and second authors and then discussed to reach a consensus. Before coding, researchers documented their preconceptions, reflecting upon and discussing how these could inform the analyses. Field notes were written to document the researchers' understanding of the data. In the third step, the codes derived from all interviews were combined into potential themes. All authors read and wrote in English, and the coding and potential themes were in English. The corresponding citations to these themes were translated into English, with care taken to maintain the accuracy, context and cultural relevance of the original Turkish data. In the fourth step, all authors met, partially face‐to‐face and partially online, to review themes and create a conceptual map. Potential themes were then redefined and structured into main themes and subthemes. During these meetings, researchers revisited their reflexive notes to identify their understanding of the coding, and these insights were discussed within the multicultural team. In the fifth step, themes were refined, clear distinctions were made and individual codes were assigned. In the final step, titles for themes and subthemes were described to align with the content, and the report was written. Themes were reviewed by the authors through an in‐depth discussion, referring to codes and revisiting raw data within each transcript, following a thematic reflexive process (Braun and Clarke 2021; Näsström et al. 2023).

### 4.5 Characteristics of Participants

Participants consist of Turkish individuals living and ageing in Norway (aged ≥ 60 years). In total, 10 of the participants are women and seven are men. Six of the 17 participants included in this study are between the ages of 60 and 64, nine are between the ages of 65 and 69 and two are between the ages of 70 and 74. All participants were married. Participants have resided in Norway for an average of 27 years (Table 1).

**TABLE 1:** Description of participants.

Participant no.	Age	Gender	Length of living in Norway	Education level	Ability to speak Norwegian
P‐1	65–69	Male	36 years	Middle school	Yes
P‐2	65–69	Male	17 years	Primary school	Yes
P‐3	70–74	Male	35 years	Middle school	Yes
P‐4	65–69	Male	13 years	Middle school	A little
P‐5	65–69	Male	35 years	Primary school	Yes
P‐6	65–69	Female	18 years	Primary school	Yes
P‐7	65–69	Female	33 years	Illiterate	Yes
P‐8	65–69	Female	33 years	Illiterate	No
P‐9	60–64	Female	26 years	Primary school	Yes
P‐10	60–64	Male	8 years	Primary school	Yes
P‐11	65–69	Male	36 years	High school	Yes
P‐12	60–64	Female	26 years	Primary school	A little
P‐13	60–64	Female	24 years	Primary school	No
P‐14	70–74	Female	33 years	Primary school	No
P‐15	60–64	Female	31 years	Primary school	A little
P‐16	65–69	Female	26 years	Primary school	Yes
P‐17	60–64	Female	29 years	Primary school	Yes

### 4.6 Ethical Considerations

This research project was registered and conducted in accordance with the protocol of the Norwegian Centre for Research Data (Ref. No. 984579). The Regional Committee for Medical Research Ethics Central Norway determined that ethical approval was not required for the study (Ref. No. 564436). Participants were provided with both written and verbal information regarding the study's purpose, their right to decline participation, the confidential treatment of interview data and their prerogative to withdraw at any time. Prior to the interviews, written informed consent was obtained from each participant.

### 4.7 Rigour

In this study, rigour was ensured through several strategies to maintain credibility, dependability, confirmability and transferability (Cypress 2017). For credibility, the researchers carefully reviewed existing literature to design a semi‐structured interview guide. This guide was utilised for data collection, and every effort was made to ensure that the participants were neither distracted nor influenced during the interviews. To enhance dependability, the analysis process was systematically conducted by multiple researchers. In line with the principles of reflexive thematic analysis, the research team encouraged each other to reflect on and acknowledge their preconceptions and positionality, using these to inform the analysis which was co‐created. An audit trail was maintained documenting each step of the data collection, analysis and interpretation. Confirmability was ensured by keeping detailed field notes and transcribing interviews verbatim, followed by cross‐checking the transcriptions against the audio recordings. Thus, notes regarding preconceptions and coding were critically reflected upon individually and further discussed in research meetings. Transferability was supported through detailed descriptions of the participants' backgrounds, the social and cultural context of Turkish immigrants living in Norway and their interactions with healthcare services. Together, these strategies ensured the trustworthiness of the study's findings. The consolidated criteria for reporting qualitative research (COREQ) was used to clearly and comprehensively report this study (Tong, Sainsbury, and Craig 2007).

## 5 Findings

Two main themes and five subthemes were identified. The first theme was ‘Utilization of the healthcare service to their best’, with the subthemes: (a) ‘Communication through a translator: Expressing health problems’ and (b) ‘From physician to physician: Seeking a second opinion in health’. The second theme was ‘Being born in Türkiye: Aging in Norway’, with the subthemes: (a) ‘Between two worlds: Efforts to establish balance’, (b) ‘Family ties and care preferences: Understanding the care preferences’ and (c) ‘Two cultures, one life: Lifestyles’. (Table 2).

**TABLE 2:** Themes and subthemes.

Themes	Subthemes
Theme 1: Utilisation of the healthcare service to their best	Communication through a translator: Expressing health problems
	From physician to physician: Seeking a second opinion in health
Theme 2: Being born in Türkiye: Ageing in Norway	Between two worlds: Efforts to establish balance
	Family ties and care preferences: Understanding the care preferences
	Two cultures, one life: Lifestyles

### 5.1 Main Theme: Utilisation of the Healthcare Service to Their Best

Participants shared their expectations, experiences and thoughts about benefiting from health services at the highest possible level. Throughout the interviews, participants noted aspects of Norway's healthcare system that were important to them. As a result of these interviews, two subthemes were found.

#### 5.1.1 Communication Through a Translator: Expressing Health Problems

This subtheme revealed that participants used different methods to express their health problems. Some participants had communication or language problems despite having lived in Norway for a long time. They had difficulty expressing themselves and preferred to express themselves through a translator (*P1*, *M*, *for 20 years*; *P9*, *F*, *for 26 years*; *P10*, *M*, *for 8 years*; *P12*, *F*, *for 26 years*; *P13*, *F*, *for 24 years*; and *P14*, *F*, *for 33 years*). One participant explained the situation as follows: *‘They hire a translator so that we can communicate. It is easy to communicate this way. We speak Turkish*, *the translator translates. I can understand everything*, *but I cannot speak very well myself’*. (*P12*, *F*, *for 26 years*). Not all participants made use of a translator when they had difficulty expressing themselves. One participant stated that when she lacked words to express herself and her symptoms or problems, she conveyed her discomfort to the general practitioner by simply showing the painful area and using signs, without saying much: *‘If my stomach hurts*, *I show my stomach*, *so they understand. If my throat hurts*, *I*'*ll show it*, *they*'*ll understand. I express it with signs’*. (*P8*, *F*, *for 33 years*). However, some female participants expressed that they could not explain their symptoms and signs to their physician at all because of language problems and being too embarrassed to present them to the translator. Instead, they hid their discomfort and were left undiagnosed and untreated for looming health problems (*P13*, *F*, *for 24 years*; and *P14*, *F*, *for 33 years*). One of the participants explained the present situation as follows: *‘A translator comes whenever I want. I cannot explain some things because I am ashamed*, *which causes me to hide them’*. (*P14*, *F*, *for 33 years*). Some participants conveyed their discomfort by using their children or spouses as a translator rather than an unknown translator and claimed this worked well, although they did not consider the concerns of using a family member as a translator (*P9*, *F*, *for 26 years*; *P13*, *F*, *for 24 years*; and *P14*, *F*, *for 33 years*). One participant expressed this situation as follows: *‘I can express this more easily when I use my children’*. (*P9*, *F*, *for 29 years*).

#### 5.1.2 From Physician to Physician: Seeking a Second Opinion in Health

The theme ‘From physician to physician: Seeking a second opinion in health’ explains patients' trust in and preferences regarding the healthcare system. The participants generally receive healthcare services from local physicians and specialists and seem satisfied with this system. However, in certain situations, especially during vacations or when they feel the need for additional confirmation, they prefer to get a second opinion from a private specialist in Türkiye. One participant expressed this situation as follows: *‘For example*, *all the tests were done here* (*Norway*), *everything is fine. Everything is clean*, *but I took my wife to a physician in İstanbul to make us feel at ease’*. (*P1*, *M*, *for 20 years*). Another participant: *‘When I took my wife to Türkiye*, *we had an x‐ray taken to see if the diagnoses here* (*Norway*) *and there* (*Türkiye*) *confirmed each other. The result is the same. Physicians here say the same things as there and apply their treatments’*. (*P4*, *M*, *for 13 years*). Not all participants experience that physicians in Türkiye and Norway recommend the same examinations for the same condition. One participant said: ‘*I went to Türkiye because of my heart condition. They told me I would need to have an angiography. When I came back to Norway*, *I told the physicians that the physicians in Türkiye had said I needed to have an angiography. However*, *the Norwegian physicians gave me medication*, *and I took it. After that*, *I did not undergo angiography’*. (*P8*, *F*, *for 33 years*).

### 5.2 Main Theme: Being Born in Türkiye: Ageing in Norway

This main theme deals with the experiences of individuals who were born in Türkiye and then immigrated to Norway, in regard to living as citizens in a new country, the difficulties they face in this process and their experiences of ageing. During the interviews, participants expressed their experiences of evaluating the opportunities being between two worlds, their family ties, care preferences and belonging to two cultures with different lifestyles.

#### 5.2.1 Between Two Worlds: Efforts to Establish Balance

The effort to establish a balance in their experience of two different worlds plays an important role in the lives of Turkish older individuals living in Norway. Although some participants sorely miss Türkiye, they adapt to a part‐time stay in Norway because their children and grandchildren are there (*P1*, *M*, *for 20 years*; *P5*, *M*, *for 35 years*; *P7*, *F*, *for 33 years*; *P10*, *M*, *for 8 years*; *P12*, *F*, *for 26 years*; and *P13*, *F*, *for 24 years*). One participant expressed this situation as follows: *‘Our plan is to live 6 months there* (*Türkiye*) *and 6 months here* (*Norway*). *I prefer this place because our children and grandchildren are here*, *so I am thinking of coming here* (*Norway*)*’*. (*P12*, *F*, *for 26 years*). They consider spending sometime in Türkiye and sometime in Norway to be a good way to keep family ties warm and avoid forgetting their roots. Most participants decided not to return to Türkiye at all in order not to lose their pensions (*P7*, *F*, *for 33 years*; and *P14*, *F*, *for 33 years*.) *‘I choose to grow old here because my salary is paid regularly and my grandchildren live here’*. (*P7*, *F*, *for 33 years*). Although they long for certain things like the nature and climate of Türkiye, advantages such as health services, living standards and security in Norway cause them to postpone the idea of moving to Türkiye (*P7*, *F*, *for 33 years*; *P14*, *F*, *for 33 years*; *P15*, *F*, *for 31 years*; and *P17*, *F*, *for 29 years*). Trying to balance the opportunities offered by both worlds, these Turkish individuals continue to stay true to their roots and benefit from the opportunities Norway offers.

#### 5.2.2 Family Ties and Care Preferences: Understanding the Care Preferences

While some participants emphasised that the health services and facilities offered in nursing homes in Norway are quite good, they stated that living in a nursing home is perceived negatively in Turkish society (*P1*, *M*, *for 20 years*; *P3*, *M*, *for 35 years*; *P5*, *M*, *for 35 years*; and *P9*, *F*, *for 26 years*). One participant explained this situation as follows: *‘Nursing homes are beautiful places*… *But we prefer not to live in a nursing home. Because this is perceived as an embarrassing situation for us. Our children do not send their parents to nursing homes*, *but the nursing homes here are more comfortable’*. (*P5*, *M*, *for 35 years*). Additionally, most participants indicated a preference for receiving care from their children rather than staying in a nursing home (*P2*, *M*, *for 17 years*; *P9*, *F*, *for 26 years*; *P11*, *M*, *for 36 years*; and *P13*, *F*, *for 24 years*). One participant stated the following: *‘I wouldn*'*t want to stay in a nursing home. I want my children to take care of me’* (*P11*, *M*, *for 36 years*).

#### 5.2.3 Two Cultures, One Life: Lifestyles

This subtheme reveals distinct differences among lifestyles, values and physical activities in Norwegian and Turkish cultures. Some participants point out that older Norwegians lead a more active life compared to older Turkish people, they prefer to live alone and encourage their children to leave home and start their own lives at the age of 18 (*P5*, *M*, *for 35 years*; *P9*, *F*, *for 26 years*; and *P15*, *F*, *for 31 years*). One participant stated: *‘Norwegians are constantly traveling*, *walking*, *running. Older people often live alone. When their children reach the age of 16‐18*, *they are encouraged to leave home and start their own lives. Even if they have many children*, *they prefer to live separately. In our culture*, *the situation is different. We are generally used to living together within the family. I would be disturbed if I couldn*'*t see my children even for a day. However*, *Norwegians prefer to live alone. Our cultural differences on this issue are clear’*. (*P9*, *F*, *for 26 years*). Additionally, family ties are emphasised more strongly in Turkish culture. Some participants stated that elements such as covering children's wedding expenses, the process of establishing a home and the support provided to children highlight the sense of solidarity and responsibility within the family (*P5*, *M*, *for 35 years*; *P9*, *F*, *for 26 years*; *P11*, *M*, *for 36 years*; and *P15*, *F*, *for 31 years*). One participant stated: *‘Norwegians tell their children*: *“Son*, *you are 18 years old*, *find a place for yourself*, *find a partner*, *get married*.” *The child is leaving too. He finds a house*, *finds a wife*, *gets married*, *starts a family. Things are different in our culture. First*, *we cover the child*'*s wedding expenses*, *then we buy a house and a car. We contribute to their budget. We give gold to the child after marriage. So we have a structure like buy this*, *buy that’*. (*P5*, *M*, *for 35 years*). These cultural differences reveal various perspectives arising from expectations, family relationships and individual priorities in old age.

In terms of physical activities, some of the female participants stated that Norwegians generally adopt an active lifestyle and participate in activities such as walking, running and travelling, while Turkish women generally fulfil household chores and religious responsibilities rather than physical activities (*P9*, *F*, *for 26 years*; *P12*, *F*, *for 26 years*; *P14*, *F*, *for 33 years*; and *P16*, *F*, *for 26 years*). One participant expressed this situation as follows: *‘I pray five times a day*, *spend time reading the Quran and go to the mosque. My children have grown up*, *now I spend time with my children and grandchildren. They visit me often and we go shopping together. I prefer to watch television while relaxing at home’* (*P9*, *F*, *for 26 years*). Some of the participants stated that they would age more actively if they lived in Türkiye (*P4*, *M*, *for 13 years*; and *P7*, *F*, *for 33 years*), while some stated that they would age more actively in Norway (*P1*, *M*, *for 20 years*; and *P3*, *M*, *for 35 years*).

## 6 Discussion

### 6.1 Communication Through a Translator: Expressing Health Problems

This study sheds light on the complex nature of communication barriers encountered by Turkish immigrants in Norway when they access healthcare services. The employment of various strategies for communication, such as professional translators, family members and nonverbal signals, emphasises the intricate process of navigating healthcare systems in a foreign language. The presence of translators plays a crucial role in bridging language gaps, yet the feelings of discomfort and embarrassment it causes for some underline the importance of adopting culturally sensitive communication methods in healthcare. Language barriers stand as a formidable obstacle to healthcare access for immigrants, aligning with findings from previous research (Czapka and Sagbakken 2016; Karim et al. 2020; Tschirhart, Diaz, and Ottersen 2019). Tschirhart et al. specifically point out the enduring nature of these language challenges in Norway, resonating with the experiences of participants in this study who resorted to alternative communication methods in the absence of professional translators (Tschirhart, Diaz, and Ottersen 2019).

This study found that participants sometimes communicated with healthcare providers through their Norwegian‐speaking spouse or child. The use of family members as translators, while possibly practical, introduces potential issues related to the accuracy of information conveyed and privacy concerns (Straiton and Myhre 2017). This strategy risks miscommunication and places undue responsibility on untrained individuals for medical interpretation, a concern that is echoed by reports on adverse experiences due to mistranslations by nonprofessionals (Arfa et al. 2020). Sharing a common language is essential in enhancing patient experiences, as it not only boosts comfort and satisfaction but also builds trust between patients and nurses, enabling a more effective expression of health needs (Ali and Johnson 2017).

These observations make a strong case for health professionals and policymakers to focus on creating intercultural health services that effectively overcome linguistic barriers. For example, artificial intelligence (AI) has the potential to revolutionise healthcare systems by enhancing the quality of services and improving patient outcomes (Mohammed, Mohammed, and Mohammed 2022). In this particular case, AI could possibly be used to translate so that patients and healthcare providers, like nurses, can communicate easily in their own languages. This, along with training nurses to understand different cultures, could enhance healthcare services significantly. Such strategies could make healthcare more accessible and sensitive to the diverse needs of the population.

### 6.2 From Physician to Physician: Seeking a Second Opinion in Health

This study reveals that Turkish immigrants in Norway may seek second opinions from private specialists in Türkiye. It is especially during visits back home that they seek further confirmation of diagnoses received by the public services in Norway. This trend underscores a combination of trust in the private Turkish healthcare system and the need for reassurance in medical diagnoses, perhaps driven by cultural familiarity in Türkiye.

Literature suggests that seeking second opinions in one's home country with the aim of confirming initial diagnoses is a common practice among immigrants, due to reasons such as previous positive experiences, language barriers or the complexity of the health issue at hand (Herrero‐Arias and Diaz 2021; Tschirhart, Diaz, and Ottersen 2019). The decision to undergo additional tests or follow‐ups back in Norway, rather than in Türkiye, indicates a strategic approach to utilising healthcare resources across two countries, leveraging the advanced medical facilities available in Norway with the cultural and linguistic alignment found in Türkiye.

### 6.3 Between Two Worlds: Efforts to Establish Balance

The findings from this study highlight a complex interplay among economic benefits, familial ties and cultural identity among Turkish individuals of retirement age living in Norway. Participants expressed a preference for living in Norway, citing the regularity of their salaries and the benefits of Norwegian citizenship, which include not just legal protections but also economic advantages. This echoes findings from research on immigrants in similar contexts, where economic stability and legal status are significant motivators for not permanently returning to their countries of origin (Hainmueller et al. 2018). The fear of losing rights and residency status upon extended absence from the host country plays a crucial role in the decision to maintain a presence in Norway.

However, beyond the economic considerations, the emotional and psychological dimensions of living between two cultures are substantial. Many older immigrants express a deep longing for their homeland, planning to divide their time between Türkiye and Norway to maintain familial connections and not lose touch with their cultural roots. This situation reflects the longing for one's homeland and sense of belonging seen in studies with older immigrants in Sweden (Osman et al. 2020).

This study illustrates that while Turkish individuals aged 60 and above in Norway navigate the economic advantages of their situation, they also grapple with feelings of loneliness and nostalgia. The desire to remain connected to Türkiye, while leveraging the opportunities in Norway, underscores a delicate balance between embracing the benefits of their host country and the inherent pull of their homeland. These findings contribute to a nuanced understanding of the immigrant experience, revealing how economic, legal and emotional factors intertwine to shape the lives of older Turkish immigrants in Norway. The effort to maintain a connection to their roots, while adapting to and benefiting from life in Norway, illustrates a broader narrative of migration, belonging and identity.

### 6.4 Family Ties and Care Preferences: Understanding the Care Preferences

This study highlights a discernible hesitancy among Turkish individuals living in Norway to opt for nursing home residency, revealing a cultural gap in perceptions of care in old age. Despite recognising the high quality of health services and facilities in Norwegian nursing homes, these individuals exhibit a pronounced preference for care within the family, a choice deeply intertwined with societal norms and the stigma of nursing home residency as an abandonment of familial duties.

Such trends are not unique to this group but have been observed in similar contexts elsewhere. For example, in another study in China, there is a preference among older individuals and Asian immigrants for living with children, a choice motivated by the desire to uphold cultural and traditional values (Zhao, Zhou, and Zhu 2021). A study with Turkish immigrants in Belgium similarly highlighted a preference for family care over nursing homes, illustrating a widespread cultural emphasis on the importance of family and community ties in elder care (De Tavernier and Draulans 2018). Indeed, the preference for family care among older adults is often driven by a desire for closeness and continuity of family life (Du et al. 2023).

The reluctance of Turkish older adults in Norway to embrace nursing home care reflects a broader cultural imperative that cherishes family connections and intergenerational solidarity. It underscores the importance of cultural sensitivity in the design and delivery of aged care services, suggesting that care options should account for not just the physical and medical needs but also the cultural, emotional and social requirements of older immigrants.

### 6.5 Two Cultures, One Life: Lifestyles

The findings of this study highlight significant cultural distinctions between Norwegian and Turkish communities, especially in terms of lifestyles, values and approaches to physical activity. Respect for children and intergenerational solidarity are especially important in the traditional Turkish family structure. Turkish adults often express their expectations that children will take care of their parents in times of need, and that they will live with their families and provide care and support to them in the future (Ten Kate, Bilecen, and Steverink 2021).

This study reveals a distinct contrast in physical activity levels between Turkish immigrants and native Norwegians, with the former engaging less frequently in activities such as walking, running and travelling. Key findings from research on Turkish female immigrants in Norway attribute this lower level of physical activity to the challenging winter conditions, which particularly discourage outdoor activities during colder months (Rieger, Terragni, and Czapka 2021). Additionally, broader studies on immigrant women in Norway highlight how seasonal variations and concerns over safety significantly reduce their participation in physical activities, as adverse weather conditions, limited daylight and icy surfaces create substantial barriers (Lorentzen and Viken 2022).

Turkish female participants in our study preferred household chores, especially housework, family care and religious activities, rather than outdoor physical activities. This trend is consistent with the findings of another study of immigrant women in Norway, which showed that the combination of work, home responsibilities and family responsibilities limit women's opportunities for outdoor physical activity (Lorentzen and Viken 2022).

These insights emphasise the intricate interplay among cultural practices, environmental conditions and social norms in shaping the physical activity patterns of Turkish immigrants in Norway. Recognising these factors is crucial for developing inclusive and culturally sensitive approaches to health promotion that address the unique needs and preferences of diverse communities.

### 6.6 Strengths and Limitations of the Work

In this study through reflexive thematic analysis, the research group reflected on and acknowledged their preconceptions and positionality, which may have contributed to accurate and meaningful findings. Team discussions, guided by reflective notes, may have played a significant role in informing the analysis and shaping the themes. Another significant strength of our study is the flexibility offered to participants to conduct interviews in two languages, Norwegian and Turkish. In addition, we allowed participants to select their preferred interview setting, whether it was their homes, a cafe or a mosque. This flexibility in location may likely contributed to participants feeling more comfortable and at ease, encouraging them to speak freely. Furthermore, while all interviews were conducted in only one region of Norway, we included participants of both genders, with varying levels of educational attainment and knowledge of the Norwegian language. Unfortunately, we did not have participants from the oldest age group (e.g., those aged 80 and above), which limits the representation of the very oldest population. Additionally, due to practical constraints, including the health concerns of older participants, they conducted only one round of interviews.

## 7 Conclusion

This study reveals experiences Turkish immigrants have with the healthcare service and ageing while living in Norway and balancing between two cultures. These findings offer a valuable perspective for healthcare providers and social workers and offer insight relevant for developing a cross‐cultural healthcare service programme. Future research may explore patterns of other immigrants' experiences with such programmes.

## Author Contributions

All authors have agreed on the final version and meet at least one of the following criteria: (1) Substantial contributions to conception and design (Anne‐Sofie HELVIK, Büşra Nur TEMÜR and Süleyman ŞAHİN), acquisition of data (Büşra Nur TEMÜR and Süleyman ŞAHİN) or analysis and interpretation of data (Süleyman ŞAHİN, Büşra Nur TEMÜR, Selma ÖNCEL, Nilgün AKSOY and Anne‐Sofie HELVIK); (2) Drafting the article or revising it critically for important intellectual content (Süleyman ŞAHİN, Büşra Nur TEMÜR, Selma ÖNCEL, Nilgün AKSOY and Anne‐Sofie HELVIK).

## Ethics Statement

This research project was registered and conducted in accordance with the protocol of the Norwegian Centre for Research Data (Ref. No. 984579). The Regional Committee for Medical Research Ethics Central Norway determined that ethical approval was not required for the study (Ref. No. 564436).

## Conflicts of Interest

The authors declare no conflicts of interest.

## Peer Review

The peer review history for this article is available at https://www.webofscience.com/api/gateway/wos/peer‐review/10.1111/jan.16763.

## Supporting information


Data S1.

Table S1.

## Data Availability

The data that support the findings of this study are available from the corresponding author upon reasonable request.
