# Supplementary material for: The Experiences of Healthcare Services and Ageing Among Older Turkish Immigrants: A Qualitative Study
Source: J Adv Nurs. 2025 Jan 22;81(10):6625–34. doi: 10.1111/jan.16763 (PMC12460959; doi:10.1111/jan.16763)
Supplement: Supplementary file 2 — Table S1. [file JAN-81-6625-s002.docx]

**Supplement Table 1:** **Interview guide**

| The interview guide and its four main questions.  1.What are your wishes and needs for caregiving in old age  (including home nursing care, nursing home care services and/or being cared for by family members etc) (whishes/needs now and in the future)?  2.What are the experiences with and needs for healthcare services in Norway  (including access, content and usefulness to specialist care at hospital level and community-based care, including GP visits, home care services and nursing care etc) (needs/whishes now and in the future)?  3.Can you compare the expected aging status if you were living in your home country?  4.Now in old age, what do you do to have a good life and age well, both physically and mentally? |
| --- |
